# Supplementary material for: High‐Throughput Discovery of Novel Cubic Crystal Materials Using Deep Generative Neural Networks
Source: Adv Sci (Weinh). 2021 Aug 5;8(20):2100566. doi: 10.1002/advs.202100566 (PMC8529451; doi:10.1002/advs.202100566)
Supplement: Supplementary file 1 — Supporting Information [file ADVS-8-2100566-s001.pdf]

© 2021 Wiley-VCH GmbH

ADVANCED SCIENCE
Open Access

Supporting Information

for *Adv. Sci.*, DOI: 10.1002/advs.202100566

High-throughput discovery of novel cubic crystal materials using deep generative neural networks

Yong Zhao, Mohammed Al-Fahdi, Ming Hu, Edirisuriya MD Siriwardane, Yuqi Song, Alireza Nasiri, and Jianjun Hu**

Supplementary file1: High-throughput discovery of novel cubic crystal materials using deep generative neural networks

Yong Zhao, Mohammed Al-Fahdi, Ming Hu,* Edirisuriya MD Siriwardane, Yuqi Song, Alireza Nasiri, and Jianjun Hu*

Supplementary Table 1. 23 element properties used for element embedding in CubicGAN

**Properties**	**Properties**	**Properties**
Atomic number	Average ionic radius	noble gas or not
Pauling electronegativity	Average cationic radius	transition metal or not
Periodic table row	Average anionic radius	post transition metal or not
Periodic table group	Sum of all ionic radii	metalloid or not
Atomic mass	Maximum oxidation state	alkali or not
Atomic radius	Minimum oxidation state	alkaline or not
Mendeleev number	Average all common oxidation states	halogen or not
Molar volume	Average all known oxidation states	

Supplementary Table 2. Hyper-parameters for training the CubicGAN Model

Hyper-parameter		Value
batch size		256
Adam optimizer	learning rate	0.00001
	β_1	0.5
	β_2	0.9
gradient penalty coefficient		10
the number of iterations of discriminator per generator iteration		5

Supplementary Table 3. Crystal prototypes existent in our training and validation sets (OQMD-TC3, MP-TC3 and ICSD-TC3) for ternary CubicGAN. There are only 8 different prototypes.

	ABC_2-225	ABC-216	ABC_3-221	AB_2C_6-225
OQMD-TC3	185170	184162	5237	1166
MP-TC3	4343	520	1410	196
ICSD-TC3	551	280	759	233

	ABC_6-225	AB_3C_3-221	AB_3C_8-221	AB_6C_6-225
OQMD-TC3	8	4	2	0
MP-TC3	36	16	23	1
ICSD-TC3	20	6	26	0

Supplementary Table 4. Existing $ABCD_6$-216 materials in databases OQMD, MP, and ICSD

Database-ID	Formula
oqmd-24074	$BaNaH_6Ir$
oqmd-24073	$NaCaH_6Ir$
icsd-262196	$BaNaH_6Ir$
icsd-51205	$NbWNO_6$
icsd-262197	$BaNaH_6Ir$
icsd-96973	$NbWNO_6$
icsd-262195	$NaCaH_6Ir$
mp-1223322	$KRbMnF_6$
mp-1182061	$BaNaH_6Ir$
mvc-14934	$CaFeWO_6$
mp-1227207	$CaEuH_6Ru$
mp-1228944	$CsRbMnF_6$
mp-1180133	$NaCaH_6Ir$

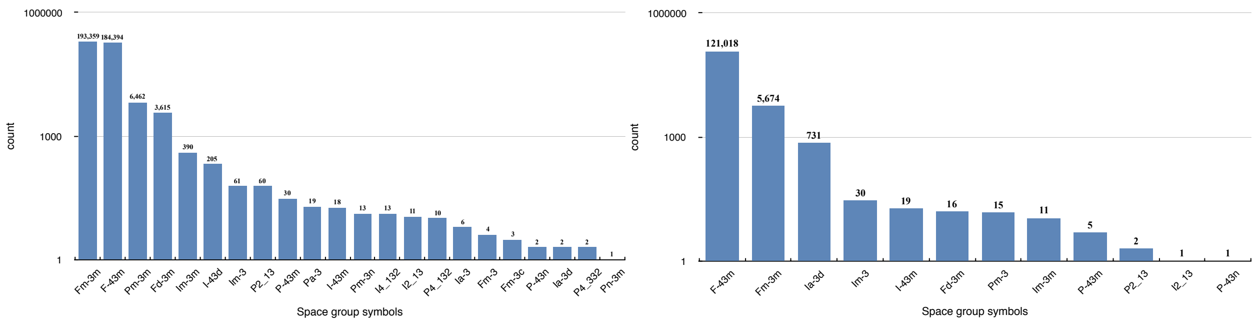

(a) Ternary (Total is 388680) (b) Quaternary (Total is 127523)

Supplementary Figure 1. Distribution of each space group of in Ternary and Quaternary Cubic systems. The top 3 cubic space groups cover the majority of known cubic materials. The bars' height is at logarithmic scale of real values.

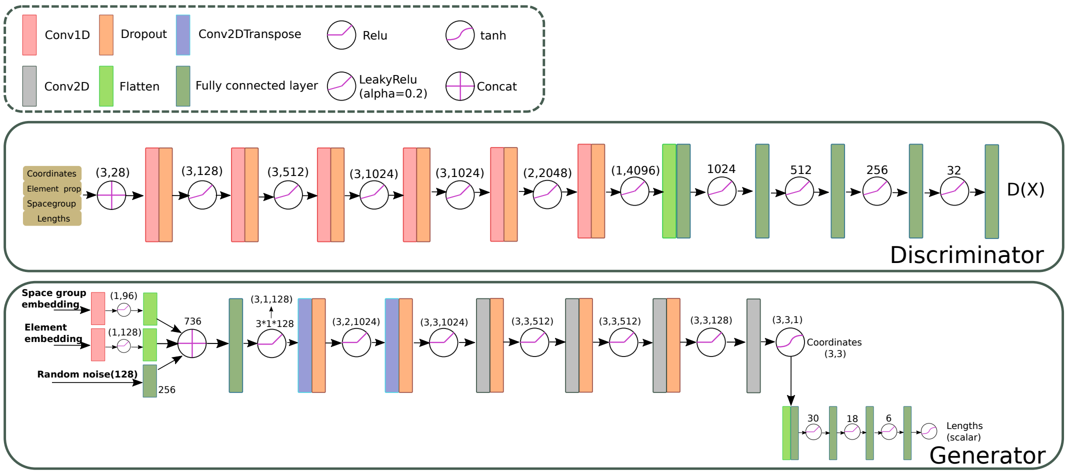

Supplementary Figure 2. The detailed architecture of the generator and the discriminator of the CubicGAN framework. Discriminator consists of 6 convolutional layers followed by Dropout layer and 5 fully connected layers. The inputs with shape (3×28) to discriminator is composed of four parts: atom coordinates and corresponding element properties, spacegroup encoding and lattice length. The inputs to Generator consist of spacegroup and element embedding and random noise vector. They are concatenated and de-convolutional layers map them to non-equivalent atom coordinates and lattice parameters.

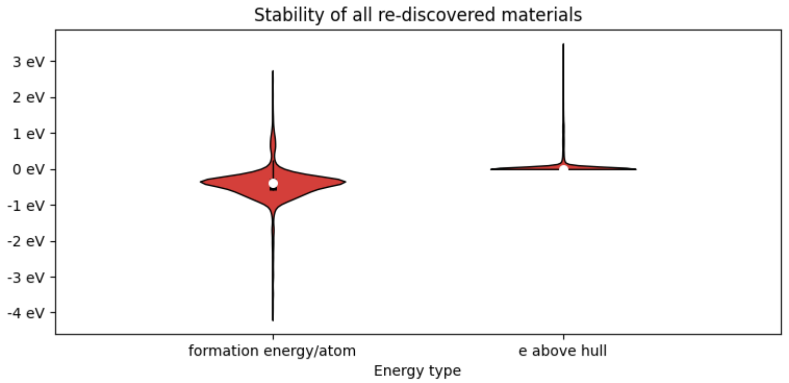

Supplementary Figure 3. Distribution of the formation energy and e-above-hull of re-discovered materials in Materials Project. When sampling ten million materials, 4731 and 950 materials are re-discovered in MP and ICSD, respectively out of all ternary cubic materials of the space groups 216/225/221 which are 6545 and 1875 for MP and ICSD. For 4731 materials in MP, we downloaded the formation energy per atom and e-above-hull energy from Materials Project [1]. We find most of these 4731 materials have negative formation energy per atom and energy-above-hull equal to zero (2521) or close to 0 eV (4346 materials' e-above-hull is below 0.2 eV) which means that most materials we re-discovered from MP are stable or meta-stable. Materials in ICSD are mostly synthesizable and experimentally determined [2]. The re-discovered materials by our method demonstrate that our method could produce stable materials that are potentially synthesizable.

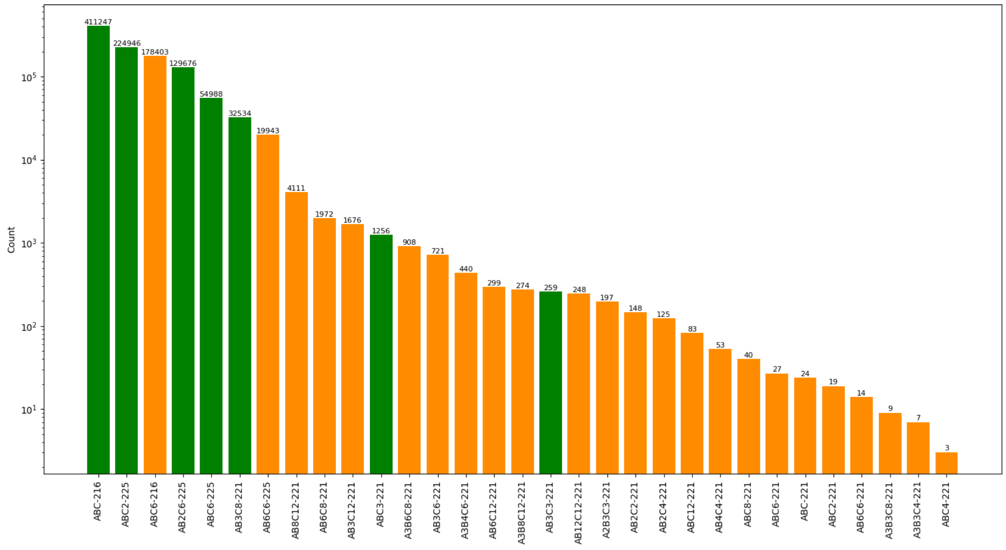

Supplementary Figure 4. Distribution of the prototypes of generated materials after removing Lanthanoid and Actinoid. Green bars show known prototypes in the training data and orange bars show the number materials of new prototypes. The figure shows that our model has generated many new prototypes. The bars' height is at logarithmic scale of real values.

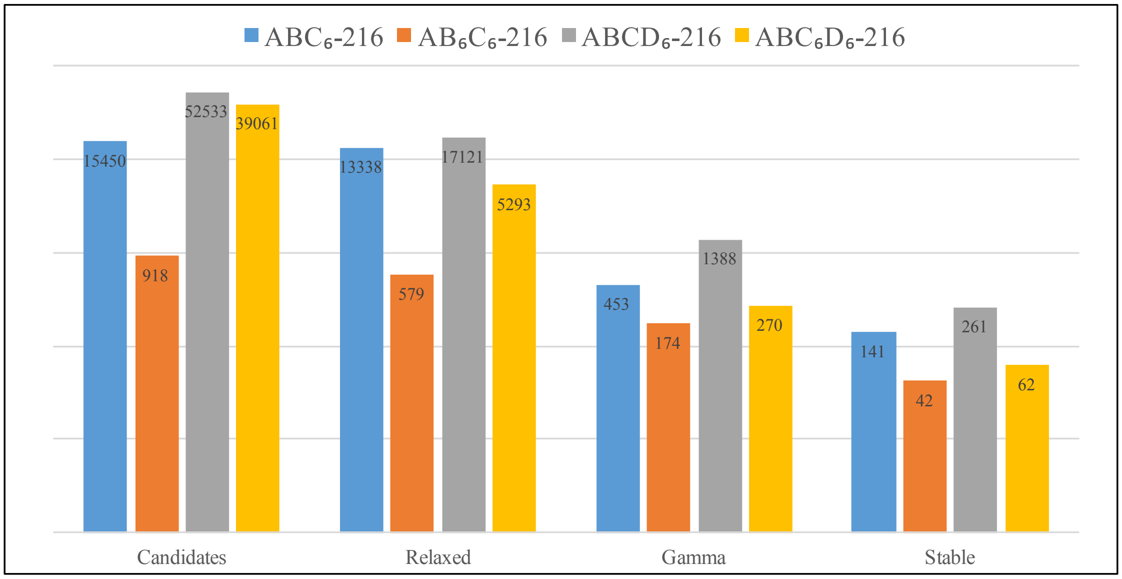

Supplementary Figure 5. The number of materials after each filtering process for four new material prototypes (ABC_6-216,AB_6C_6-225,$ABCD_6$-216,ABC_6D_6-216) (The bars' height is at logarithmic scale of real values.). We find that most of the generated structures can be successfully relaxed using DFT calculation. *Candidates* are generated structures that are charge neutral and have negative formation energy as predicted by CGCNN. *Relax* are candidate materials successfully optimized by DFT. *Gamma* are optimized structures that have positive vibrational frequencies at the $Gamma$ point, indicating the structures are potentially stable. *stable* are the final stable structures with full positive phonon dispersions as verified by DFT.

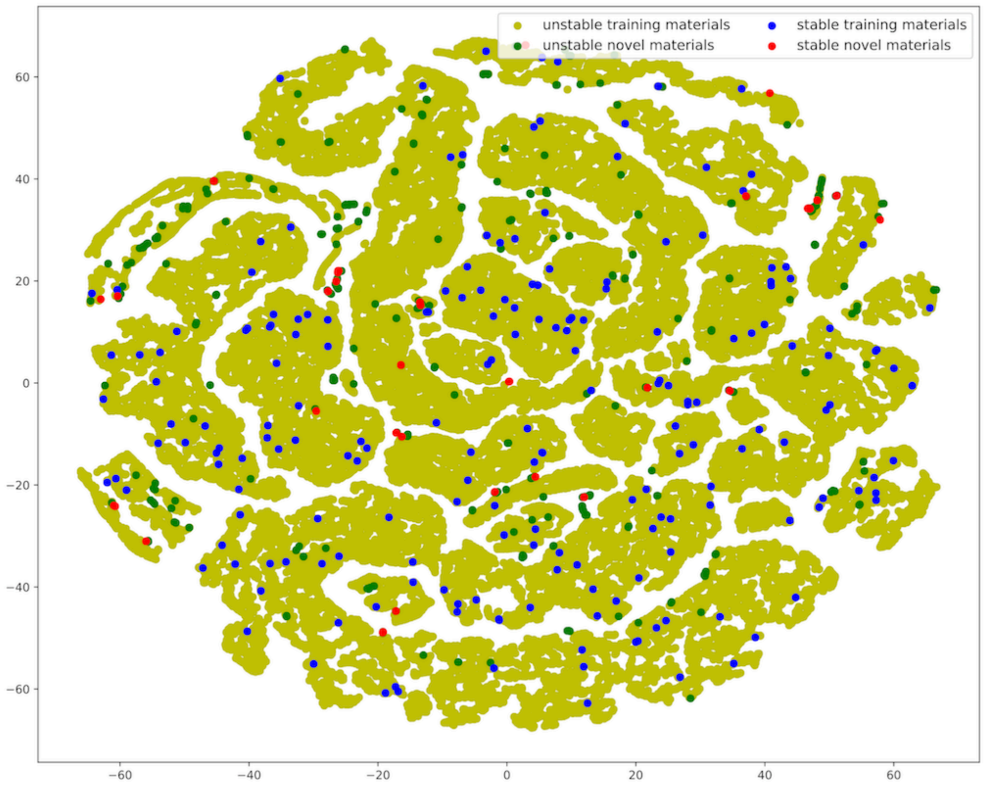

Supplementary Figure 6. Visualization of the distributions of materials in the training set and the new prototype AB_6C_6 materials both with space group of Fm$\bar{3}$m. The two dimension coordinates of each point is mapped by t-SNE from the high-dimensional XRD representations of the materials. We find that the new-prototype (AB_6C_6) materials are mostly located at the peripheral regions of know materials clusters, indicating their structural closeness to known structures, which is different from other new-prototype materials as shown in supplementary Figure 7 and Figure 8.

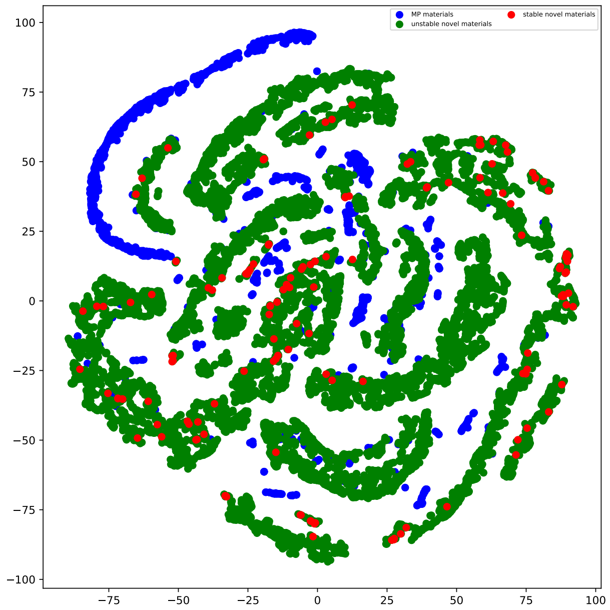

(a) Distribution of materials in MP-TC3 validation set and new ABC_6 materials with space group F$\bar{4}$3m.

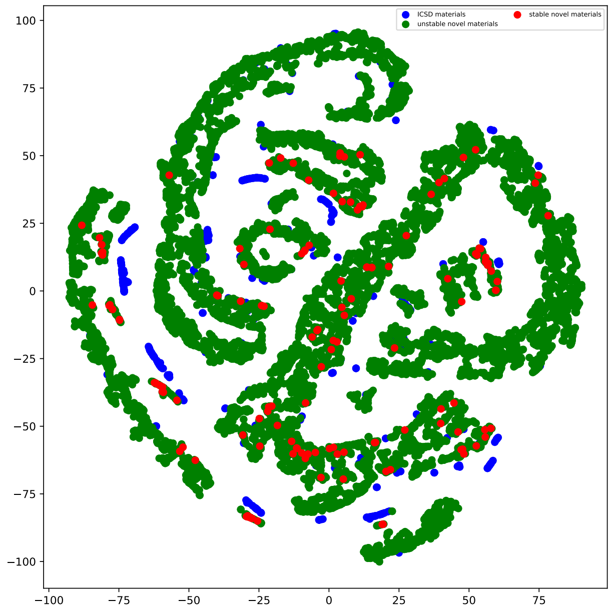

(b) Distribution of materials in ICSD-TC3 validation set and new ABC_6 materials with space group F$\bar{4}$3m.

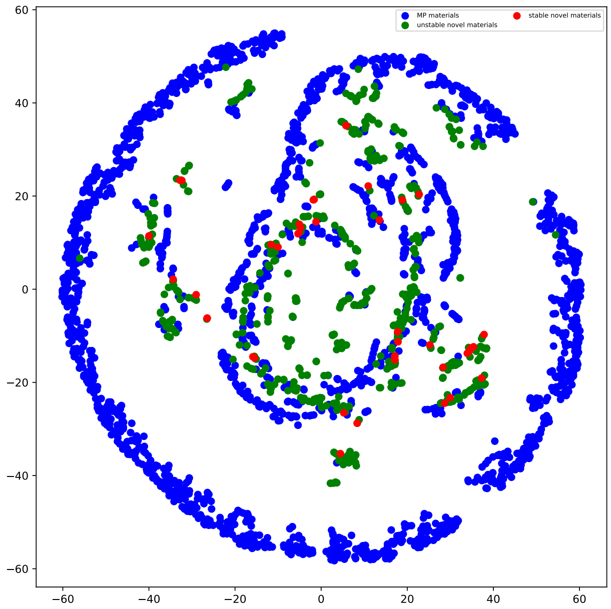

(c) Distribution of materials in MP-TC3 validation set and new AB_6C_6 materials with space group Fm$\bar{3}$m.

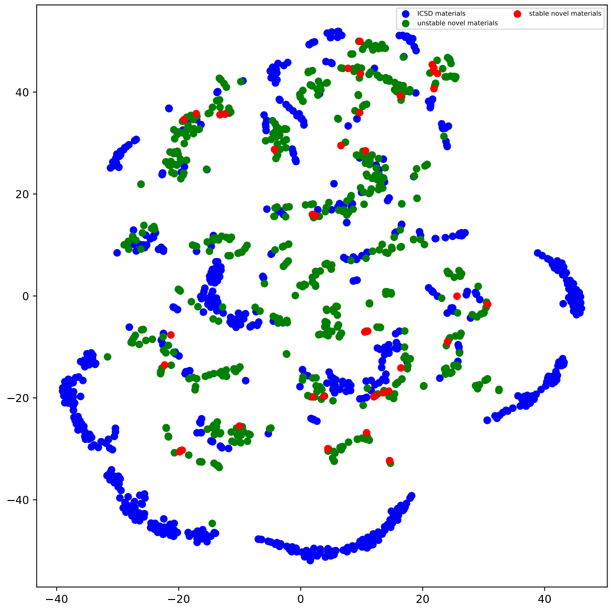

(d) Distribution of materials in ICSD-TC3 validation set and new AB_6C_6 materials with space group Fm$\bar{3}$m.

Supplementary Figure 7. Visualization of the distributions of materials in the MP-TC3/ICSD-TC3 validation sets and the new prototype (ABC_6 and AB_6C_6) materials both with space group of F$\bar{4}$3m and Fm$\bar{3}$m. The two dimension coordinates of each point is mapped by t-SNE from the high-dimensional XRD representations of the materials. We find that materials of these two new-prototypes tend to form distinct clusters indicating their structural deviation from known materials. Additionally, for most of the new-prototype clusters, we have identified one or more DFT-verified stable materials (the red points)

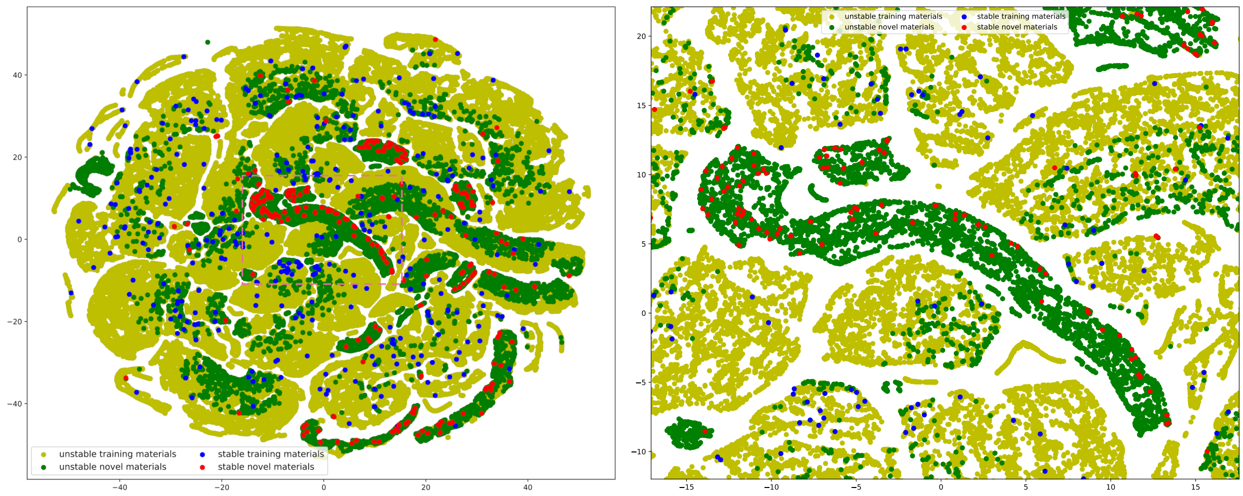

(a) Distribution of training set samples and the new-prototype $ABCD_6$ materials with space group of F$\bar{4}$3m.

(b) Local zoomed region.

Supplementary Figure 8. Visualization of the distributions of materials in the training data and the new-prototype $ABCD_6$ materials both with space group of F$\bar{4}$3m. The two dimension coordinates of each point is mapped by t-SNE from the high-dimensional XRD representations of the materials. (a) shows the overall clustering. (b) is the zoomed region as marked in main figure (a). We find for this prototype, there are multiple new clusters, each of which contains multiple DFT-verified stable materials.

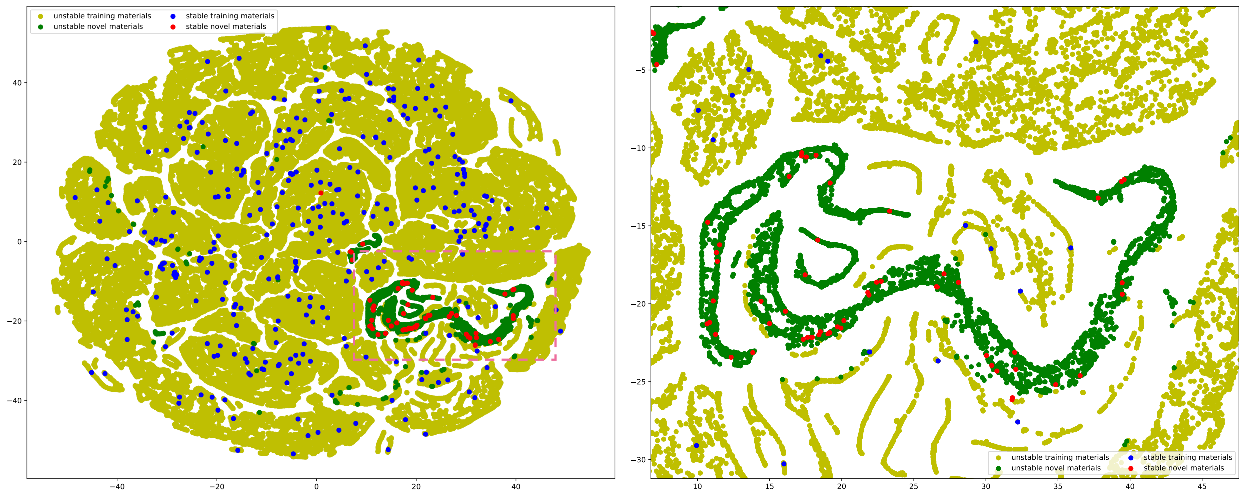

(a) Distribution of training set samples and the new-prototype ABC6D6 materials with space group of F$\bar{4}$3m.

(b) Local zoomed region.

Supplementary Figure 9. Visualization of the distributions of materials in the training data and the new-prototype ABC_6D_6 materials both with space group of F$\bar{4}$3m. The two dimension coordinates of each point is mapped by t-SNE from the high-dimensional XRD representations of the materials. (a) shows the overall clustering. (b) is the zoomed region as marked in main figure (a). For this prototype, there exist only three main new clusters.

References

[1] A. Jain, S. P. Ong, G. Hautier, W. Chen, W. D. Richards, S. Dacek, S. Cholia, D. Gunter, D. Skinner, G. Ceder, et al., *Apl Materials* **2013**, *1*, 1 011002.

[2] G. Bergerhoff, I. Brown, F. Allen, et al., *International Union of Crystallography, Chester* **1987**, *360* 77.
