# Supplementary material for: High‐Throughput Discovery of Novel Cubic Crystal Materials Using Deep Generative Neural Networks
Source: Adv Sci (Weinh). 2021 Aug 5;8(20):2100566. doi: 10.1002/advs.202100566 (PMC8529451; doi:10.1002/advs.202100566)
Supplement: Supplementary file 2 — Supporting Information [file ADVS-8-2100566-s002.pdf]

Supplementary information 2: High-throughput discovery of novel cubic crystal materials using deep generative neural networks Here, we show 100 exemplary stable materials generated by our CubicGAN. We show 25 structures for each prototype and 10 exemplary phonon dispersions. You can visit Carolina Materials Database for more details (www.carolinamatdb.org).

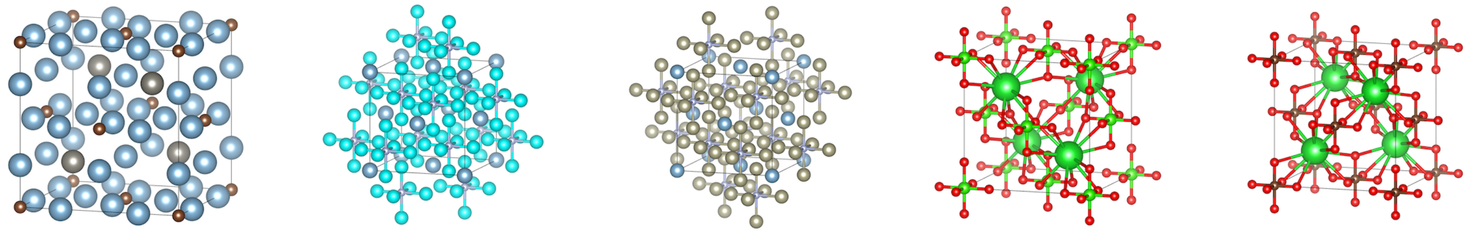

Al_6WC $AlNi_6N$ $AlRe_6N$ $BaClO_6$ $BaCO_6$

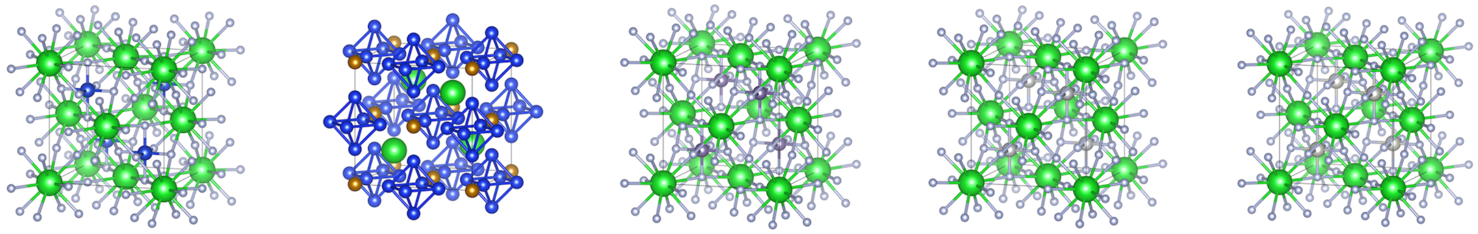

$BaCuF_6$ $BaFeSi_6$ $BaGeF_6$ $BaNiF_6$ $BaPdF_6$

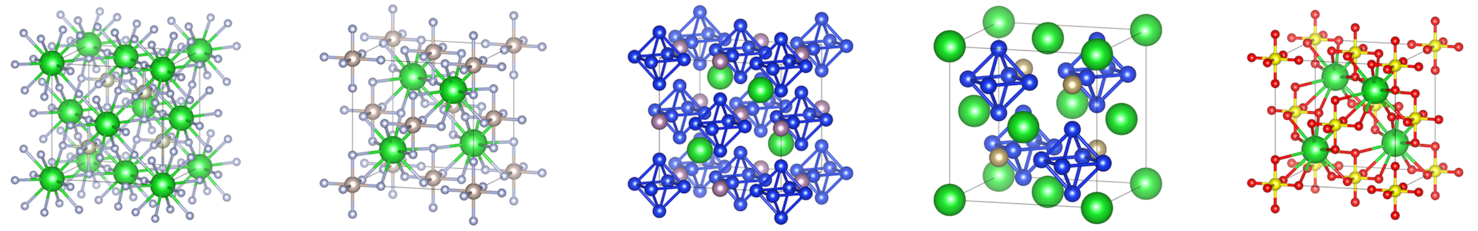

$BaRhF_6$ $BaRuF_6$ $BaSi_6Mo$ $BaSi_6Os$ $BaSO_6$

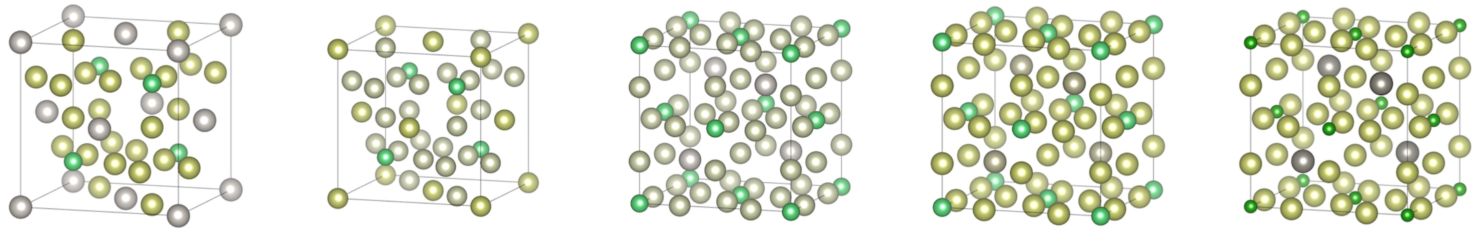

$BeIr_6Pt$ $BeIrRh_6$ $BePtRh_6$ $BeReIr_6$ BIr_6W

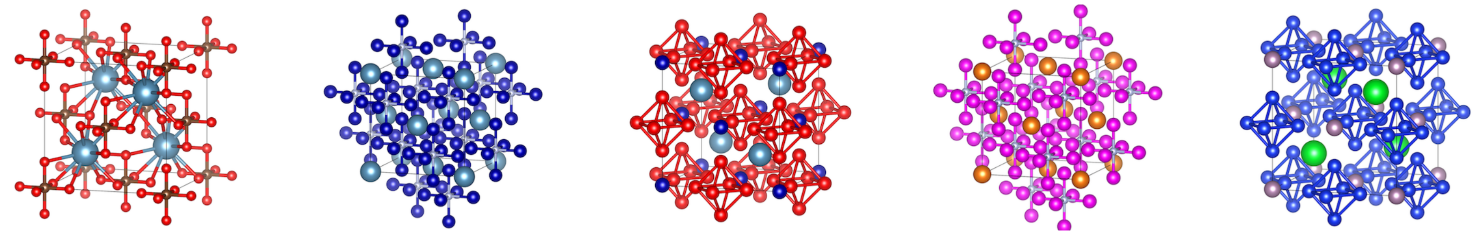

$CaCO_6$ $CaCo_6N$ $CaCoSi_6$ $MgNi_6N$ $SrSi_6Mo$

Figure 1: 25 exemplary stable materials belonging to ABC_6-216

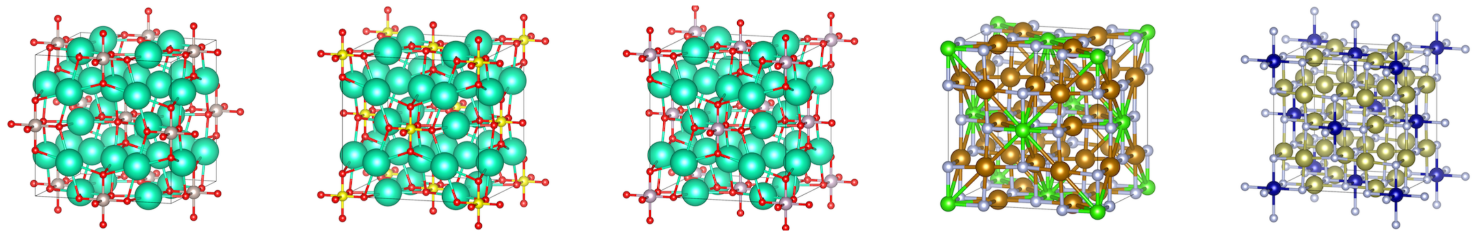

Cs_6RuO_6 Cs_6SO_6 Cs_6TcO_6 Fe_6N_6Cl Hf_6CrN_6

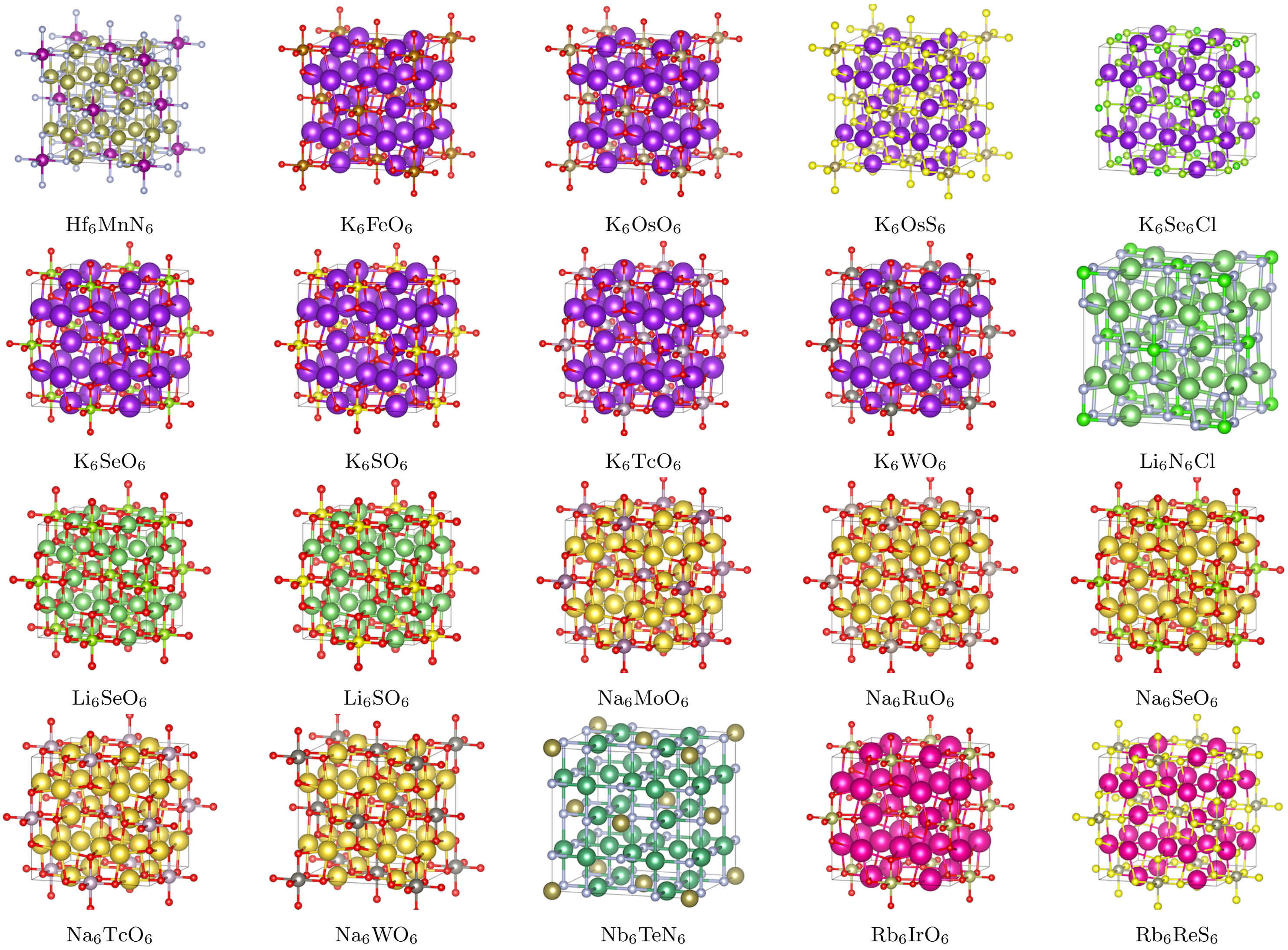

Figure 2: 25 exemplary stable materials belonging to AB_6C_6-225

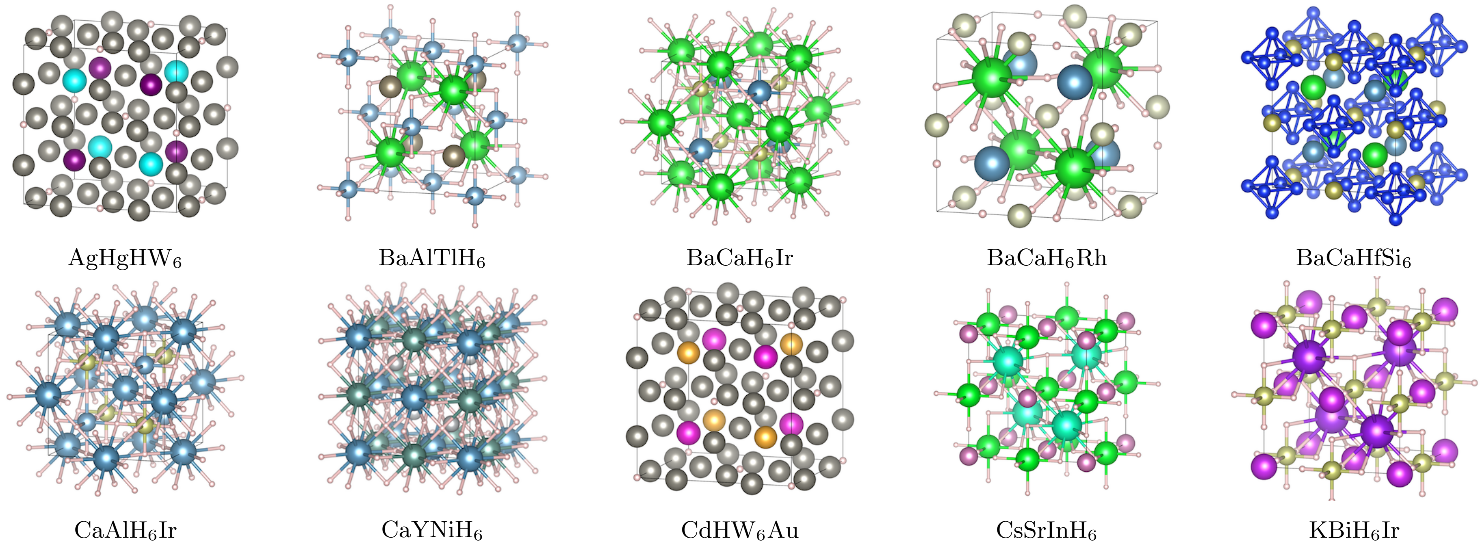

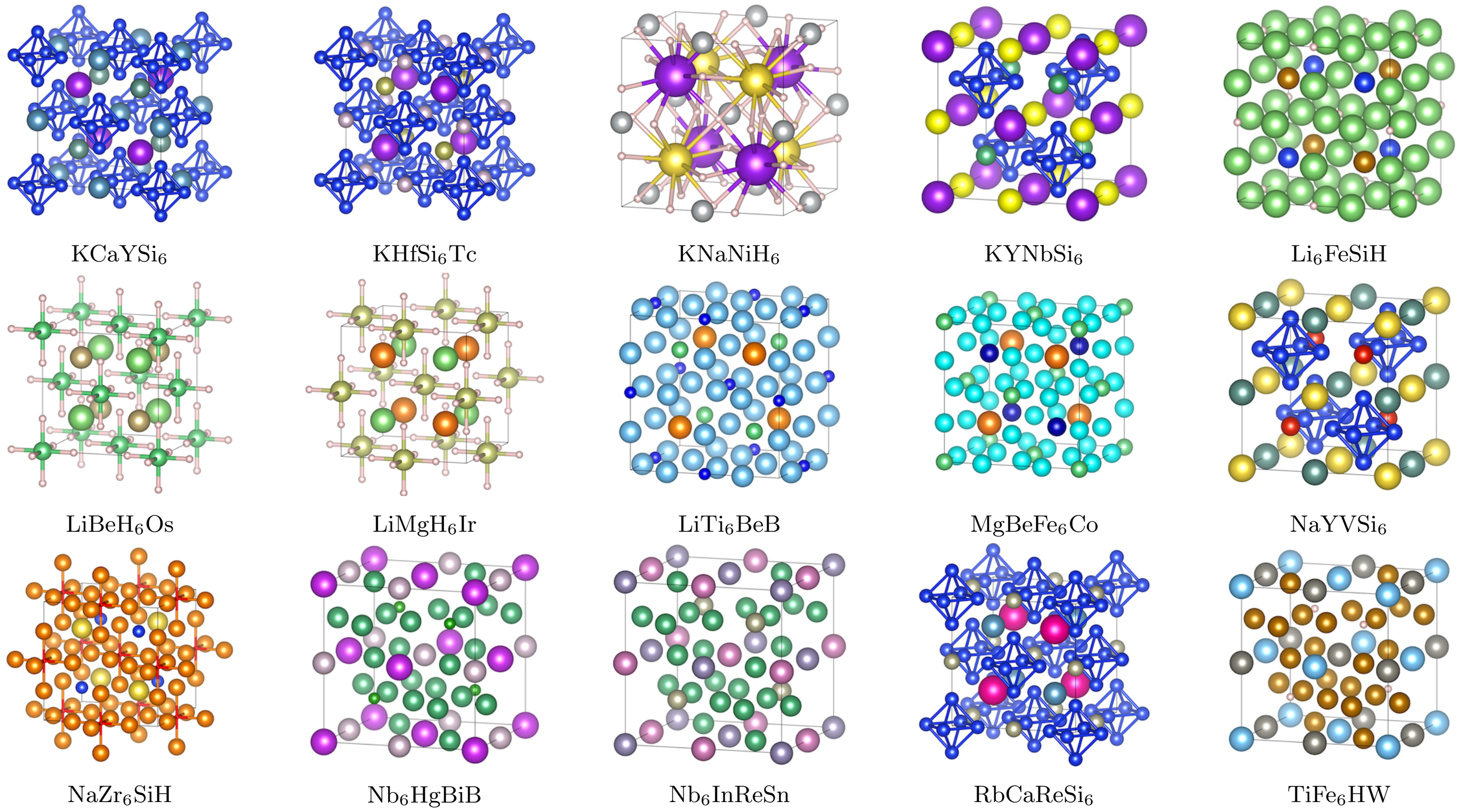

Figure 3: 25 exemplary stable materials belonging to $ABDC_6$-216

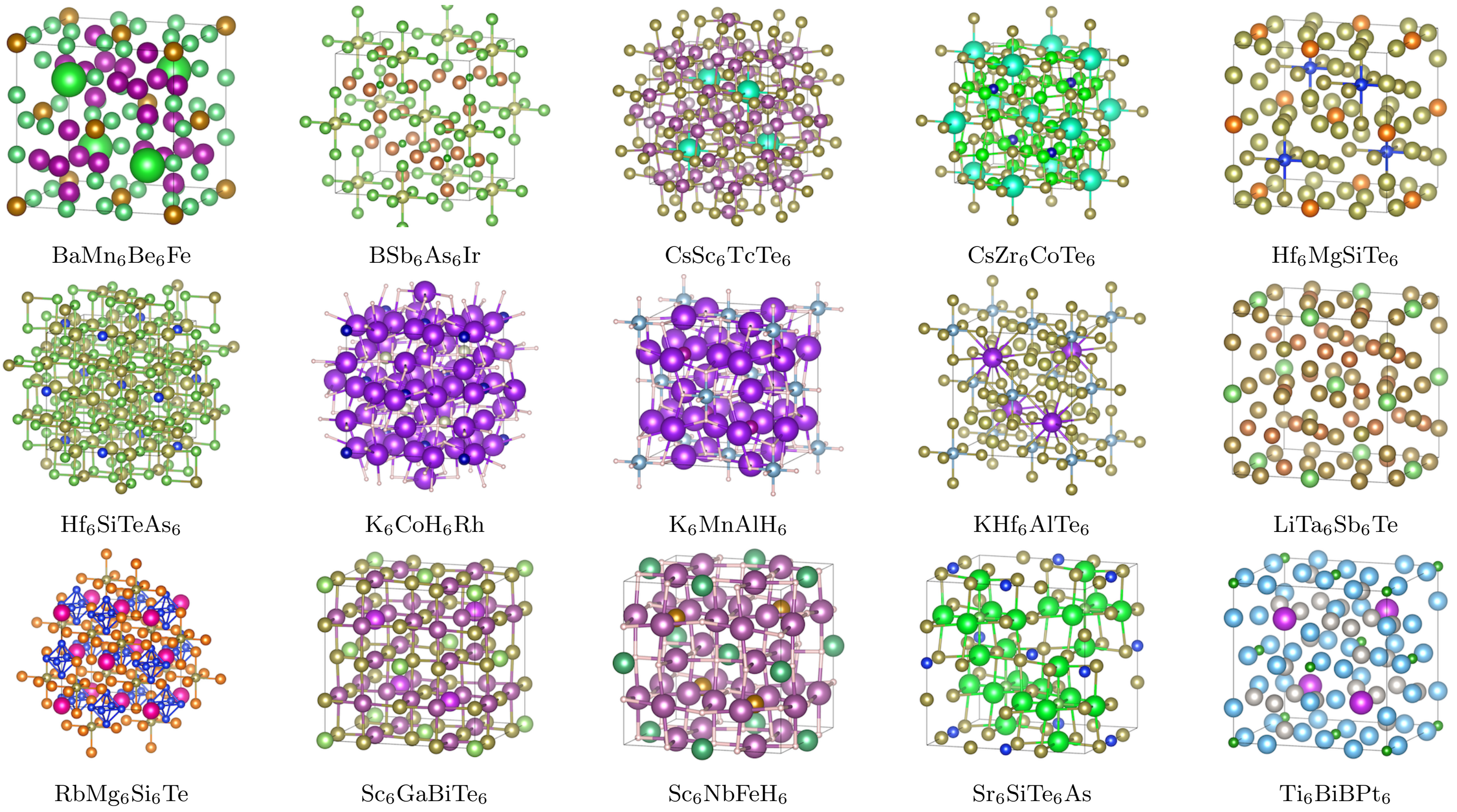

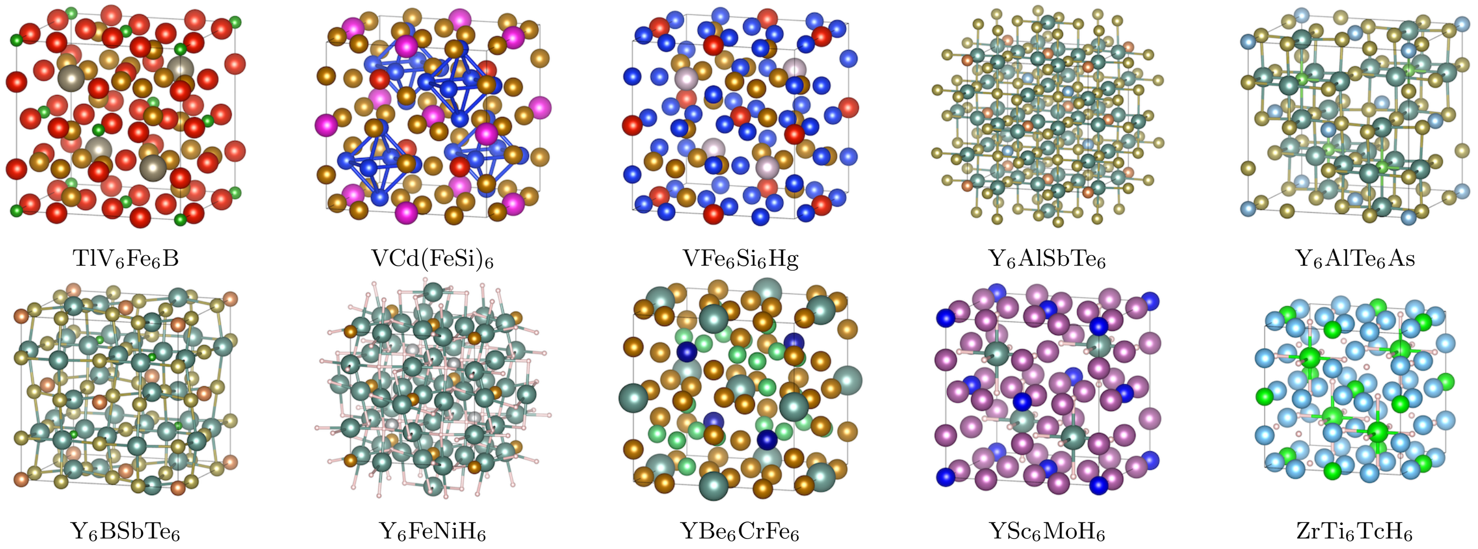

Figure 4: 25 exemplary stable materials belonging to ABC_6D_6-216

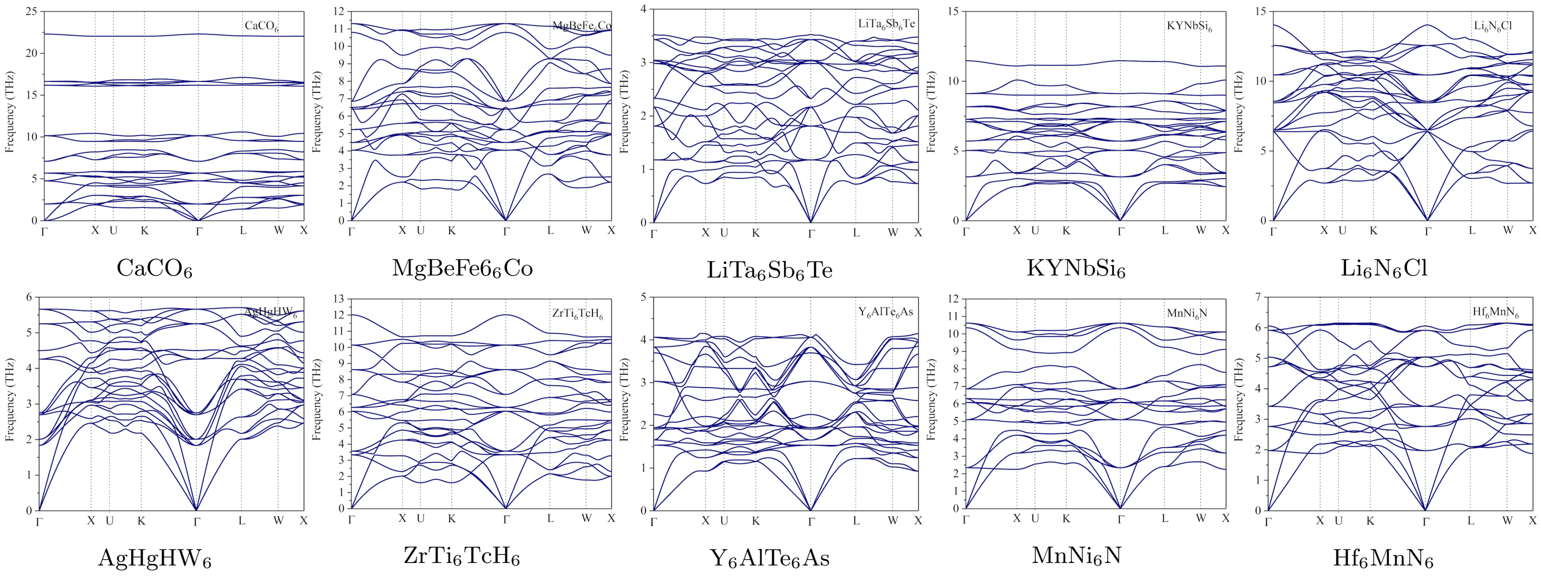

Figure 5: phonon dispersion of 10 generated materials
